# THE EFFECTS OF NEIGHBORHOOD, NETWORK, AND INDIVIDUAL EDUCATION LEVELS ON COGNITIVE HEALTH

**DOI:** 10.1093/geroni/igae098.1166

**Published:** 2024-12-31

**Authors:** Noah Webster, Laura Zahodne, Toni Antonucci, Kristine Ajrouch

**Affiliations:** University of Michigan, Ann Arbor, Michigan, United States; University of Michigan, Ann Arbor, Michigan, United States; University of Michigan, Ann Arbor, Michigan, United States; University of Michigan, Ann Arbor, Michigan, United States

## Abstract

It is well established that greater education is associated with better cognitive health. Less is known about the cognitive effects of education across levels of influence, including neighborhood, interpersonal, and individual levels. In this study we compare the effects of individuals’ education, their social network members’ education, and their neighborhood’s education on cognitive health five years later. Respondents age 60 and older who participated in Wave 3 (2015) of the longitudinal Social Relations Study based in Metro Detroit and also completed interviews in 2021 for the Detroit Area Wellness Network COVID-19 Supplement project were selected for analysis (n=161). In 2015, respondents were asked to report completed years of education and the education level of up to 10 social network members. Household addresses were geocoded and census tract educational attainment was obtained from the American Community Survey. In 2021, cognitive assessments were administered via telephone, and a latent variable measuring global cognition was derived from episodic memory, language, attention, and working memory tests. In separate models controlling for age and gender, respondents’ own education and the proportion of social network members with a bachelor’s degree, but not the proportion of people in the census tract with a bachelor’s degree, were associated with better cognitive health. In a combined model, only having a greater proportion of social network members with a bachelor’s degree remained associated with better cognitive health. Findings highlight the salience of educational resources embedded within social networks for cognitive health and point to the need for meso-level interventions.

